# Profiles and influencing factors of pulmonary fibrosis associated with biologic and conventional disease-modifying antirheumatic drugs for autoimmune diseases: a disproportionality study based on FAERS and VigiAccess

**DOI:** 10.3389/fphar.2025.1604895

**Published:** 2025-09-25

**Authors:** Xinyue Zhang, Xinhui Wu, Cheng Luo, Chun Yang, Nan Jia, Jiayi He, Qian Chen, Fei Wang

**Affiliations:** School of Clinical Medicine, Chengdu University of Traditional Chinese Medicine, Affiliated Hospital of Chengdu University of Traditional Chinese Medicine, Chengdu, China

**Keywords:** disease-modifying antirheumatic drugs, biologic drugs, DMARDs, autoimmune diseases, pulmonary fibrosis, pharmacovigilance, disproportionality analysis

## Abstract

**Background:**

Pulmonary fibrosis is a severe and potentially fatal adverse event, and its association with disease-modifying antirheumatic drugs (DMARDs) has raised long-standing concerns. However, systematic investigations on this topic are lacking. This study aims to analyze the drug-specific safety signals, characteristics, and potential patient risk factors of DMARDs-related pulmonary fibrosis.

**Methods:**

We extracted reports of pulmonary fibrosis events related to 36 types of DMARDs from the U.S. FDA’s adverse event reporting system (FAERS) and WHO-VigiAccess databases. Full database-wide and active-comparator restricted disproportionality analyses were conducted to identify the strength of safety signals for different DMARDs. Multivariable logistic regression was used to analyze risk factors for pulmonary fibrosis events associated with DMARDs.

**Results:**

In FAERS, 4,869 cases of pulmonary fibrosis were reported among 2,456,021 adverse event reports involving DMARDs. Similarly, VigiAccess documented 4,847 pulmonary fibrosis cases out of 3,488,917 DMARD adverse events. Methotrexate (reporting odds ratio in VigiAccess [ROR _VigiAccess_] = 4.39, 95% CI: 4.11–4.70), leflunomide (ROR _VigiAccess_ = 3.26, 95% CI: 2.75–3.86), sulfasalazine (ROR _VigiAccess_ = 2.33, 95% CI: 1.91–2.84), rituximab (ROR _VigiAccess_ = 1.43, 95% CI: 1.27–1.61), and tocilizumab (ROR _VigiAccess_ = 1.28, 95% CI: 1.08–1.51) consistently showed significant disproportionality signals across both databases, suggesting a potential safety concern for pulmonary fibrosis. Multivariable analysis identified older age (>65 years) as a strong and consistent risk factor across all DMARD classes, while the influence of sex varied by drug. The time to onset of pulmonary fibrosis differed significantly across different DMARDs, with B-cell inhibitors showing the shortest onset (median: 113 days, IQR = 54–397) and TNF-α inhibitors the longest (median: 523 days, IQR = 143–1,185).

**Conclusion:**

This study revealed varying degrees of pulmonary fibrosis signals related to DMARDs, with significantly overreporting observed in certain conventional and biologic DMARDs. Age was identified as a key susceptibility factor. As the use of these agents expands, clinicians should remain vigilant in monitoring for pulmonary fibrosis.

## Introduction

Disease-modifying antirheumatic drugs (DMARDs) are cornerstone therapies for managing autoimmune diseases such as rheumatoid arthritis, systemic lupus erythematosus, and psoriatic arthritis (Fugger et al., 2020; Faison et al., 2024). By suppressing immune overactivation and reducing joint inflammation, these agents significantly improve quality of life for patients (Kerschbaumer et al., 2022; Sen et al., 2024). DMARDs are classified into three categories based on their mechanisms of action, including conventional synthetic DMARDs (csDMARDs), biologic DMARDs (bDMARDs), and targeted synthetic DMARDs (tsDMARDs) (Smolen et al., 2022). These drugs exert their effects through various pathways, including inflammation modulation and immune system suppression. CsDMARDs like methotrexate act through nonspecific mechanisms, such as inhibiting immune cell proliferation, cytokine release, and folate metabolism (Zhao et al., 2022). BDMARDs and tsDMARDs target specific immune pathways, offering more precise immunomodulation.

Despite their therapeutic benefits, DMARDs carry substantial risks of adverse events (Sepriano et al., 2022). The potential pulmonary toxicity of these drugs has not been elucidated, and clinicians often face challenges in weighing the risks and benefits of treatment. Drug-induced pulmonary fibrosis is a severe adverse event characterized by progressive scarring of lung tissue (Rajan et al., 2023). Clinically, patients present with symptoms such as shortness of breath, dry cough, and irreversible decline in lung function, which may lead to respiratory failure in severe cases (Podolanczuk et al., 2023). Previous evidence has reported possible risks of pulmonary fibrosis associated with certain DMARDs, including methotrexate and biologics such as adalimumab, etanercept, and infliximab (Roubille and Haraoui, 2014; Dawson et al., 2021). However, the existing data derive largely from small case reports, showing conflicting results and a lack of systematic perspectives. Moreover, there is still no data on whether newly available DMARDs are linked to pulmonary fibrosis.

As a result, the association between DMARDs and pulmonary fibrosis remains an area with significant knowledge gaps and uncertainties. Many DMARDs share overlapping mechanisms with the pathophysiology of pulmonary fibrosis, suggesting a potential biological link. For instance, TNF-α, a key mediator of lung remodeling in fibrosis (Maranatha et al., 2022; Adel et al., 2024; Saha and Talwar, 2024), is directly targeted by some DMARDs. Similarly, immune cell dysregulation, including T-cell and B-cell modulation, influence the progression and severity of pulmonary fibrosis (Ali et al., 2021; Prêle et al., 2022; Mendoza et al., 2023). Additionally, the lack of systematic data on the pulmonary fibrosis events across different DMARDs has led to unclear monitoring guidelines in clinical practice. The international pharmacovigilance databases, FDA Adverse Event Reporting System (FAERS) and WHO-VigiAccess, contain millions of individual safety reports that can capture rare or delayed adverse event signals. These databases are particularly useful for identifying low-incidence but high-severity events (Conte et al., 2019; Davis et al., 2020). Disproportionality analyses, using algorithms like the reporting odds ratio (ROR) or information component (IC), allow for quantifying the strength of drug-event associations across large populations (Van Puijenbroek et al., 2002; Bate and Evans, 2009).

This study utilizes the FAERS and VigiAccess databases to systematically evaluate disproportionate associations between different categories of DMARDs and pulmonary fibrosis. We identified drugs with significant pulmonary fibrosis signals across DMARD types, characterized high-risk populations, and analyzed time-to-onset patterns. The results of this study are expected to provide valuable pharmacovigilance insights for rheumatologists in managing autoimmune patients and contribute to the more precise application of DMARDs in clinical practice.

## Materials and methods

### Guidelines

We followed the 2024 Reporting of A Disproportionality Analysis for Drug Safety Signal Detection Using Individual Case Safety Reports in Pharmacovigilance (READUS-PV) guidelines (Fusaroli et al., 2024). This pharmacovigilance study utilized individual case safety reports from the FAERS database and aggregated data from the VigiAccess. The primary analysis employed disproportionality algorithms to investigate the extent of disproportionate reporting of pulmonary fibrosis events associated with various DMARDs.

### Data sources and pre-processing

The FAERS database contains a large amount of individual-level safety report data, managed by the FDA, and is designed to detect potential safety issues with all FDA-approved drugs after they are marketed (Dhodapkar et al., 2022). FAERS receives safety reports for FDA-approved products from around the world. We extracted all 20,755,634 safety reports from FAERS for the period from January 2004 to December 2023, spanning 80 consecutive quarters. For cases with multiple versions of follow-up reports, we retained the most recent version. To exclude potential duplicate cases, we applied filters based on key variables, including age, gender, country of occurrence, drug, adverse event, and event date. For reports with identical key variables, only one report was retained. To exclude reports where pulmonary fibrosis might have been caused by other factors, we further filtered out cases involving the use of bleomycin, a known drug that causes pulmonary fibrosis and is used to construct the animal model of pulmonary fibrosis (Liu et al., 2017). Additionally, we excluded cases involving concomitant medications used to treat existing pulmonary fibrosis, including nintedanib and pirfenidone.

VigiAccess is a web-based tool launched by the World Health Organization. It allows users to access aggregated safety profiles and the number of specific adverse events from the VigiBase, the WHO global database of adverse event reports for medicines and vaccines developed and maintained by Uppsala Monitoring Centre (Shankar, 2016). We retrieved the VigiAccess on 10 December 2024 for all reported DMARDs-related pulmonary fibrosis events.

### Drugs of interest and identification of adverse events

To provide a comprehensive assessment, we included routine and emerging DMARDs for autoimmune diseases as far as possible. Specifically, we considered the following six categories of DMARDs: csDMARDs (methotrexate, leflunomide, sulfasalazine, hydroxychloroquine, azathioprine, cyclosporine, mycophenolate mofetil, cyclophosphamide, penicillamine, sodium aurothiomalate, auranofin), TNF-α targeted inhibitors (etanercept, infliximab, adalimumab, golimumab, certolizumab pegol), B-cell inhibitors (belimumab, rituximab), T-cell inhibitors (abatacept), interleukin inhibitors (anakinra, canakinumab, guselkumab, Ixekizumab, risankizumab, sarilumab, secukinumab, tocilizumab, ustekinumab, tildrakizumab, brodalumab), JAK and related DMARDs (tofacitinib, baricitinib, upadacitinib, iguratimod, filgotinib), and PDE4-targeted inhibitor (apremilast). Since the “DRUGNAME” and “PROD_AI” information provided by FAERS is not always standardized drug name, we also used the DrugBank (https://go.drugbank.com/) to identify synonyms and brand names for each DMARD of interest. For example, for etanercept, we included additional search terms such as “Benepali”, “Enbrel”, “Erelzi”, “Eticovo”, “Nepexto” in the “DRUGNAME” and “PROD_AI” fields of FAERS files. In the VigiAccess, drugs are already standardized according to their active ingredients, so we used the generic names of the DMARDs for our searches.

All adverse events in FAERS and VigiAccess were structured according to the Medical Dictionary for Regulatory Activities (MedDRA) Preferred Terms (PTs). PT terms “pulmonary fibrosis” and “idiopathic pulmonary fibrosis” were used to identify pulmonary fibrosis events. To enhance the specificity of safety signal detection, only reports where the DMARD of interest was listed as the primary suspect drug were considered. Furthermore, in FAERS, we included DMARD users and those who reported indications contained any PTs under the MedDRA HLGT category “Autoimmune disorders”, in order to balance the comparators as much as possible. In VigiAccess, since individual-level indication data were not available, we included all reports involving DMARDs use.

### Statistical methods

We first performed a descriptive analysis of DMARDs-related pulmonary fibrosis, exploring trends over time and geographical distribution. For each reported case, we collected baseline characteristics including age, sex, country of occurrence, and reporter type. To handle missing values for age, sex, and country, we applied the random forest-based imputation method using the missRanger R package (version 2.6.1) with default parameters, which has shown good performance for both binary and continuous variables (Wright and Ziegler, 2017). We also presented the percentage of missing data for each variable (Table 1).

**TABLE 1:** Characteristics of cases with disease-modifying antirheumatic drugs drug-related pulmonary fibrosis.

Characteristic	Overall, N = 4,869	TNF-α-targeted inhibitors, N = 1,989	Conventional DMARDs N = 1,339	PDE4-targeted inhibitor, N = 19	B-cell inhibitors, N = 589	Interleukin inhibitors, N = 405	JAK inhibitors, N = 259	T-cell inhibitors, N = 269
Age (years)								
≤45	314 (9.96%)	97 (7.76%)	118 (11.45%)	1 (8.33%)	24 (10.53%)	43 (17.00%)	11 (5.91%)	20 (10.36%)
45–65	1,368 (43.39%)	554 (44.32%)	466 (45.20%)	6 (50.00%)	92 (40.35%)	98 (38.74%)	69 (37.10%)	83 (43.01%)
>65	1,471 (46.65%)	599 (47.92%)	447 (43.36%)	5 (41.67%)	112 (49.12%)	112 (44.27%)	106 (56.99%)	90 (46.63%)
Unknown	1,716	739	308	7	361	152	73	76
Gender								
Female	2,864 (65.16%)	1,236 (65.92%)	777 (61.81%)	10 (58.82%)	174 (51.03%)	269 (69.51%)	183 (72.33%)	215 (81.13%)
Male	1,531 (34.84%)	639 (34.08%)	480 (38.19%)	7 (41.18%)	167 (48.97%)	118 (30.49%)	70 (27.67%)	50 (18.87%)
Unknown	474	114	82	2	248	18	6	4
Country								
Canada	1,624 (36.36%)	307 (17.77%)	619 (49.64%)	2 (10.53%)	323 (55.40%)	183 (47.66%)	46 (19.33%)	144 (53.73%)
United states	1,083 (24.24%)	645 (37.33%)	177 (14.19%)	10 (52.63%)	44 (7.55%)	67 (17.45%)	103 (43.28%)	37 (13.81%)
United Kingdom	461 (10.32%)	211 (12.21%)	125 (10.02%)	0 (0.00%)	75 (12.86%)	27 (7.03%)	2 (0.84%)	21 (7.84%)
Germany	242 (5.42%)	94 (5.44%)	84 (6.74%)	0 (0.00%)	33 (5.66%)	14 (3.65%)	6 (2.52%)	11 (4.10%)
Argentina	146 (3.27%)	85 (4.92%)	3 (0.24%)	0 (0.00%)	0 (0.00%)	2 (0.52%)	47 (19.75%)	9 (3.36%)
France	133 (2.98%)	35 (2.03%)	56 (4.49%)	2 (10.53%)	22 (3.77%)	13 (3.39%)	3 (1.26%)	2 (0.75%)
Brazil	123 (2.75%)	86 (4.98%)	1 (0.08%)	0 (0.00%)	13 (2.23%)	12 (3.13%)	0 (0.00%)	11 (4.10%)
China	75 (1.68%)	25 (1.45%)	14 (1.12%)	0 (0.00%)	17 (2.92%)	5 (1.30%)	11 (4.62%)	3 (1.12%)
Japan	62 (1.39%)	19 (1.10%)	31 (2.49%)	0 (0.00%)	1 (0.17%)	5 (1.30%)	5 (2.10%)	1 (0.37%)
Denmark	58 (1.30%)	20 (1.16%)	30 (2.41%)	0 (0.00%)	7 (1.20%)	1 (0.26%)	0 (0.00%)	0 (0.00%)
Sweden	58 (1.30%)	30 (1.74%)	10 (0.80%)	4 (21.05%)	8 (1.37%)	3 (0.78%)	1 (0.42%)	2 (0.75%)
Others	402 (9.00%)	171 (9.90%)	97 (7.78%)	1 (5.26%)	40 (6.86%)	52 (13.54%)	14 (5.88%)	27 (10.07%)
Unknown	402	261	92	0	6	21	21	1
Reporter								
Health-professional	3,190 (67.83%)	1,117 (58.76%)	1,026 (80.91%)	17 (89.47%)	487 (83.53%)	286 (70.62%)	90 (34.88%)	167 (62.08%)
Consumer	1,513 (32.17%)	784 (41.24%)	242 (19.09%)	2 (10.53%)	96 (16.47%)	119 (29.38%)	168 (65.12%)	102 (37.92%)
Unknown	166	88	71	0	6	0	1	0

DMARDs, disease-modifying antirheumatic drugs; JAK, janus kinase; TNF, tumor necrosis factor; PDE4, phosphodiesterase 4.

Next, we used two classical disproportionality methods to determine whether a safety signal for pulmonary fibrosis was present in each DMARD: the reporting odds ratio (ROR) and the information component (IC) algorithms. The calculations are described below:

$$ROR=\frac{A*D}{C*B}$$

$$IC=\log2\frac{\;N_{observed}}{N_{expected}\;}$$

Here, *A* represents the number of pulmonary fibrosis reports among patients using the target DMARD, while *B* is the number of pulmonary fibrosis reports among patients not using this DMARD. *C* indicates reports of other adverse events among users of the target DMARD, and *D* represents reports of other adverse events among non-users. *N*_*observed*_ is the actual number of pulmonary fibrosis events observed among DMARD users (*A*), and *N*_*expected*_ is the expected count, calculated as (*A + B*) (*A + C*)/(*A + B + C + D*). The ROR method was selected due to its straightforward interpretation and proven effectiveness in detecting adverse drug event signals (Rothman et al., 2004). The IC approach, derived from Bayesian neural network, reduces false positive findings often seen in small sample scenarios.

A positive drug safety signal was defined by meeting the routine pharmacovigilance thresholds: (a) the lower limit of the 95% confidence interval (CI) for ROR >1, (b) the lower limit of the 95% credibility interval for IC > 0, and (c) at least three reported pulmonary fibrosis events. Only drugs that met the above criteria simultaneously were considered to have a significantly disproportionate reporting signal for pulmonary fibrosis.

To further confirm these findings, we also used an active comparator-restricted design. Typically, pharmacovigilance analyses use the full database as the control group (full database-wide disproportionality analysis), which may have baseline imbalances between cases and non-cases. Therefore, we performed further analyses within a restricted population that reported autoimmune disease co-morbidities. Cases with autoimmune diseases were identified using MedDRA PTs (version 27.0) within the High-Level Group Term category “autoimmune disorders” and/or by treatment with DMARDs. In the FAERS analysis, both full database-wide and active comparator-restricted disproportionality analyses were conducted. The VigiAccess-based analyses were restricted to those treated with DMARDs due to limited access to individual-level data.

Moreover, we explored the influence of age and sex on pulmonary fibrosis reporting across DMARD classes using FAERS data. Univariable and multivariable logistic regression analyses were applied, adjusting for reporting year, region, age (in the sex analysis), and sex (in the age analysis). To assess the timing of pulmonary fibrosis onset after drug initiation, we constructed time-to-onset plots for each DMARD category, excluding extreme outliers below the 2.5th percentile or above the 97.5th percentile. Differences in time-to-onset among DMARD classes were analyzed using the Kruskal-Wallis test, followed by Dunn’s post-hoc multiple comparison test with Bonferroni correction for significant results (*P* < 0.05). R version 4.3.1 was used for statistical analysis and visualization.

## Results

### Participants

**FIGURE 1:**
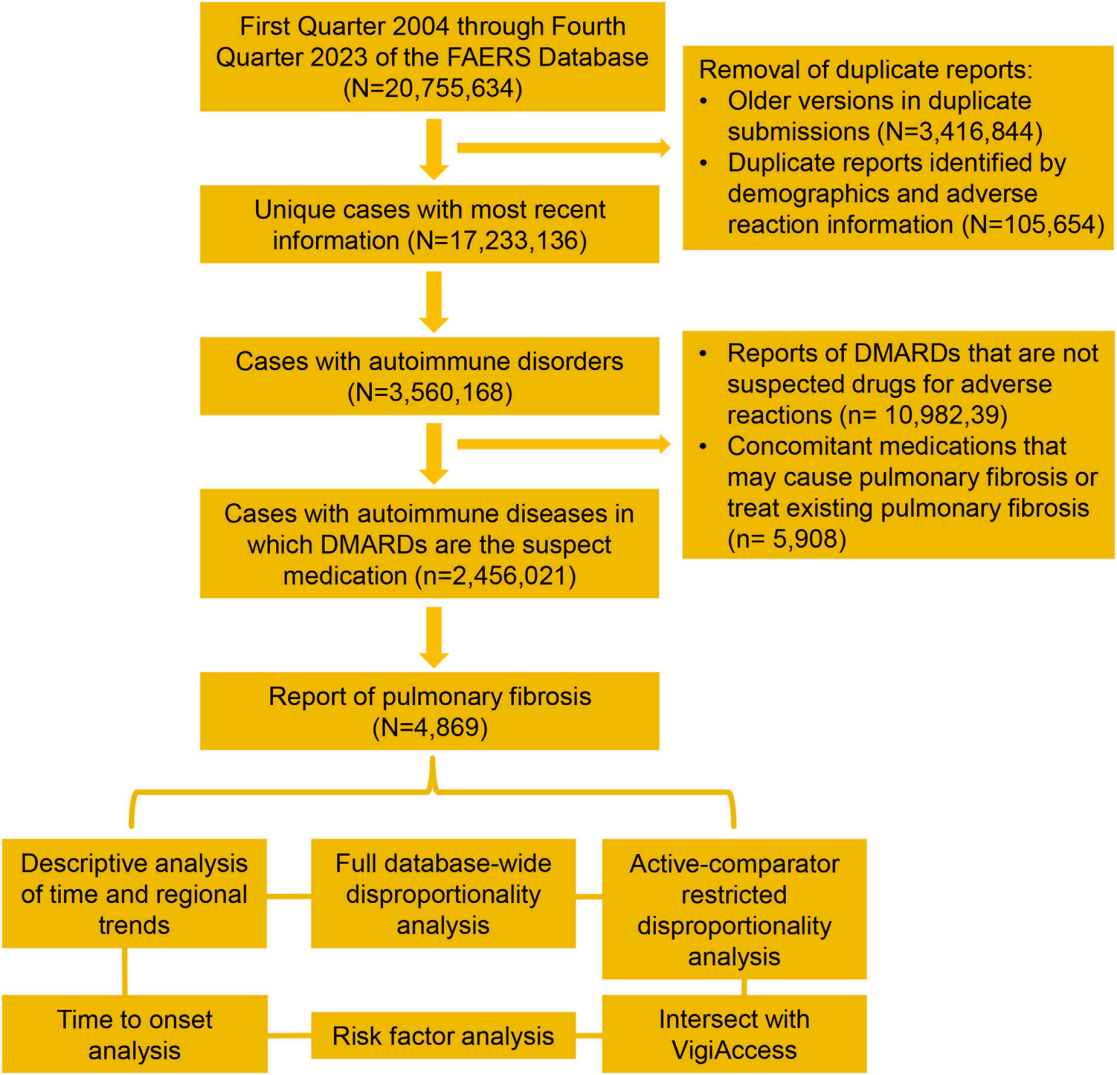
Data processing and screening workflow for the safety reports of FAERS. FAERS, Food and Drug Administration’s Adverse Event Reporting System, DMARDs, disease-modifying antirheumatic drugs.

From 2004 to 2023, FAERS received a total of 20,755,634 safety reports. After removing duplicates, 17,233,136 unique reports were included in the analysis, which represents the study population used in full database-wide analyses. We identified 3,560,168 cases with autoimmune diseases, of which 2,456,021 reports indicated DMARDs as the primary suspected drug for adverse events, including 4,869 reports of pulmonary fibrosis. A flowchart of the case screening process is shown in Figure 1. For VigiAccess, 3,488,917 reports of DMARD-related adverse events were included, of which 4,847 were pulmonary fibrosis.

### Descriptive analysis

Among the 4,869 pulmonary fibrosis events related to DMARDs in FAERS, 1989 were associated with TNF-α inhibitors, representing 40.85% of the total events. CsDMARDs were the second most reported group (n = 1,339, 27.50%), followed by B-cell inhibitors (n = 589, 12.10%), interleukin inhibitors (n = 405, 8.32%), T-cell inhibitors (n = 269, 5.52%), JAK inhibitors (n = 259, 5.32%), and PDE4-targeted inhibitor (n = 19, 0.39%). The majority of cases involved patients over the age of 45 (n = 2,839, 90.04%), with a higher proportion of female patients (n = 2,864, 65.16%). The countries reporting the most cases were Canada (n = 1,624, 36.36%), followed by the United States (n = 1,083, 24.24%) and the United Kingdom (n = 461, 10.32%). Table 1 provides a specific description of the characteristics of pulmonary fibrosis cases associated with different DMARDs.

**FIGURE 2:**
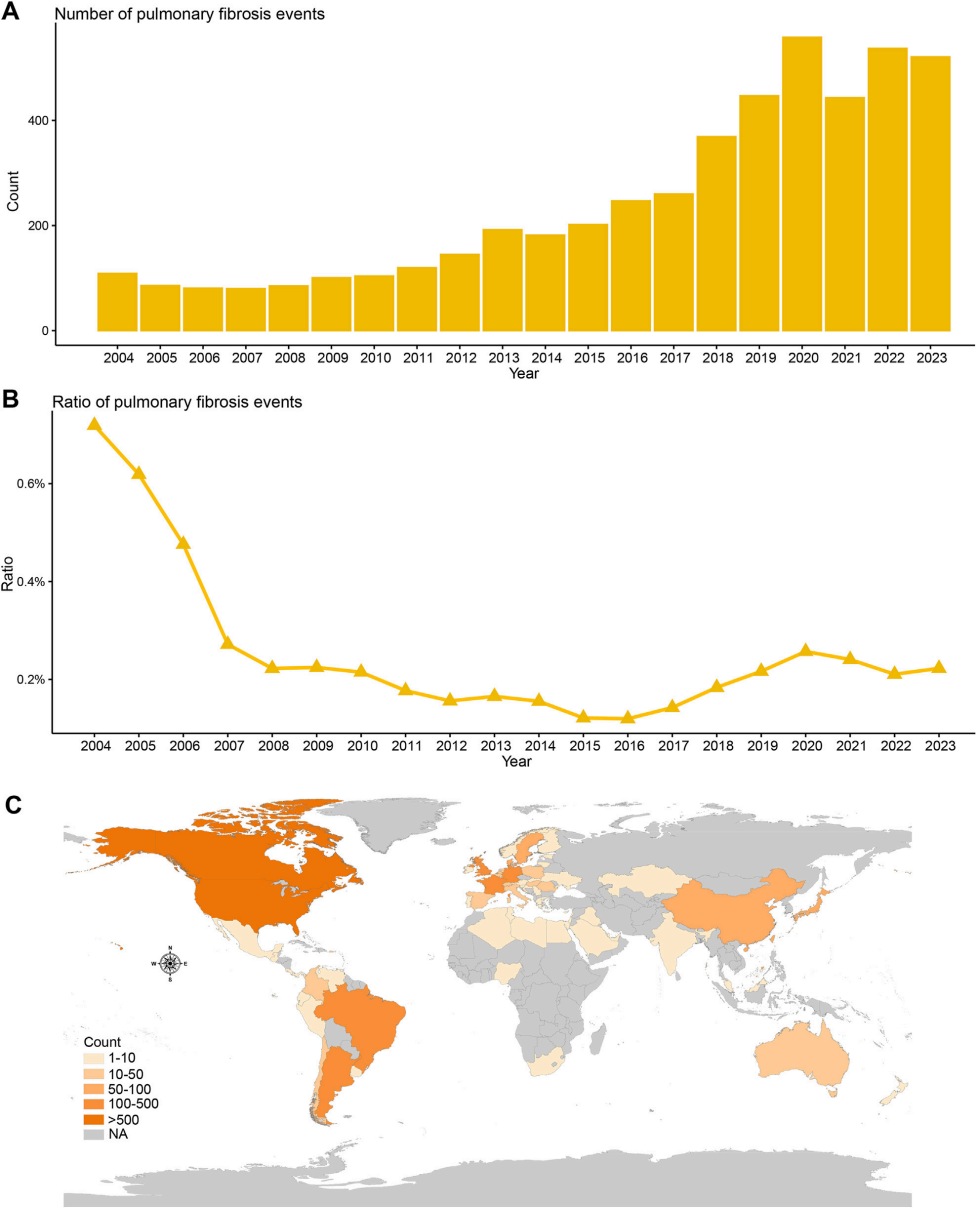
Temporal and geographical distribution trends of DMARDs-related pulmonary fibrosis in FAERS. **(A)** Number of unique DMARDs-related pulmonary fibrosis cases from January 2004 to December 2023. **(B)** Proportion of DMARDs-related pulmonary fibrosis cases among all DMARDs safety cases from January 2004 to December 2023. **(C)** Geographical distribution of DMARDs-related pulmonary fibrosis cases by country/region.

When examining the trend over time, pulmonary fibrosis cases related to DMARDs appeared to increase, from around 100 cases in 2004 to 500 cases in 2023 (Figure 2A). Interestingly, the proportion of pulmonary fibrosis among all DMARD-related adverse events has declined (Figure 2B). Most of the cases were reported from the Americas (Figure 2C).

### Full database-wide disproportionality analysis in FAERS

**FIGURE 3:**
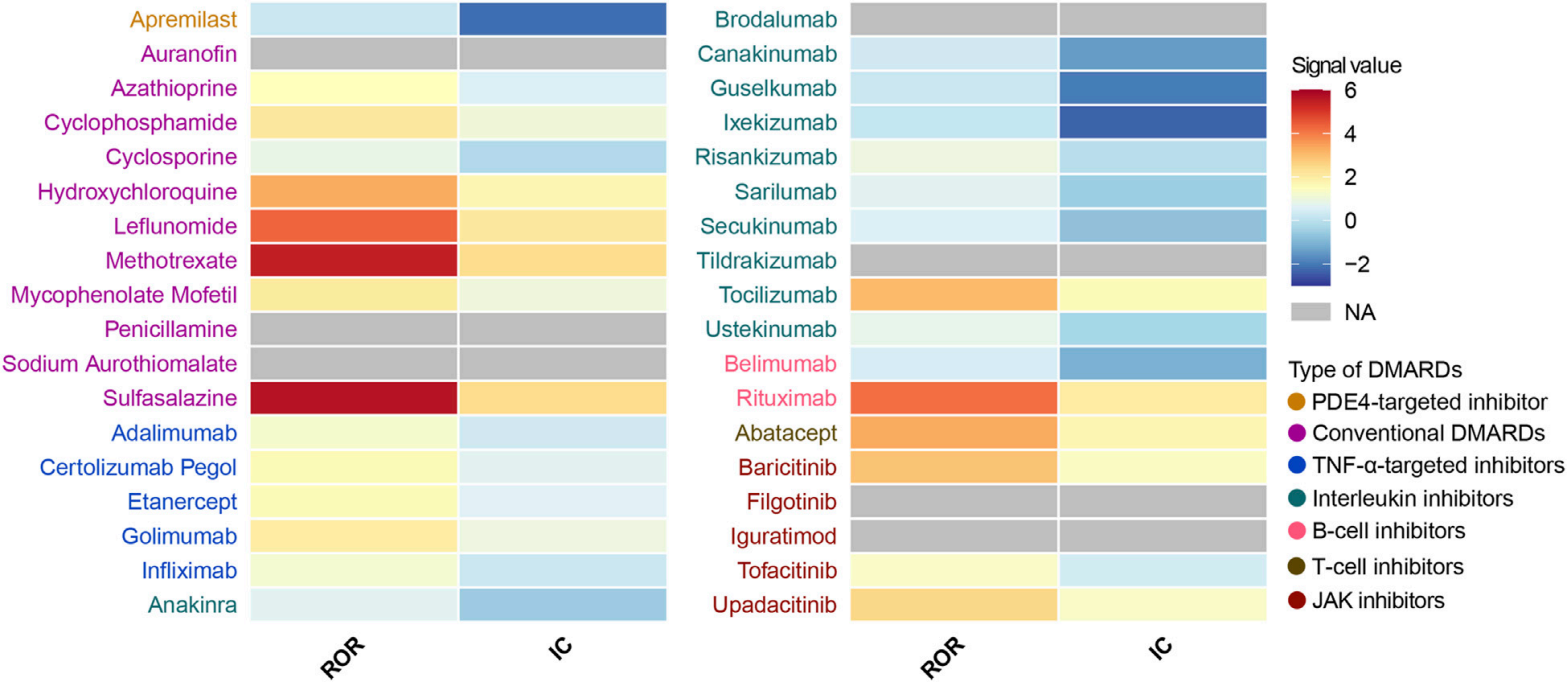
Heatmap presents full database-wide disproportionality results of pulmonary fibrosis safety signals for 36 DMARDs in FAERS. *NA* indicates that the number of reported cases is fewer than 3. DMARDs, disease-modifying antirheumatic drugs; ROR, reporting odds ratio; IC, information component; TNF, tumor necrosis factor; JAK, Janus kinase. NA, not available.

We performed a disproportionality analysis to assess the strength of the pulmonary fibrosis signal for different DMARDs (Figure 3). Compared with the full FAERS database, we found that csDMARDs including sulfasalazine (ROR = 5.65, 95% CI: 4.20–7.60), methotrexate (ROR = 5.43, 95% CI: 5.07–5.82), and leflunomide (ROR = 4.31, 95% CI: 3.70–5.01) exhibited the strongest safety signals. Other csDMARDs like hydroxychloroquine (ROR = 3.30, 95% CI: 2.60–4.19), mycophenolate mofetil (ROR = 2.05, 95% CI: 1.68–2.51), and cyclophosphamide (ROR = 2.13, 95% CI: 1.65–2.76) also showed positive signals for pulmonary fibrosis. Furthermore, all TNF-α inhibitors exhibited an over-reporting signal for pulmonary fibrosis, including adalimumab (ROR = 1.26, 95% CI: 1.18–1.35), etanercept (ROR = 1.55, 95% CI: 1.44–1.67), infliximab (ROR = 1.20, 95% CI: 1.07–1.35), golimumab (ROR = 1.97, 95% CI: 1.56–2.49), and certolizumab pegol (ROR = 1.63, 95% CI: 1.35–1.96). Notably, there was a significant difference in pulmonary fibrosis signals between the two approved B-cell inhibitors. Rituximab showed a high level of over-reporting (ROR = 4.14, 95% CI: 3.82–4.50), while belimumab did not exhibit this phenomenon (ROR = 0.46, 95% CI: 0.25–0.86). For interleukin inhibitors, only tocilizumab (targeting IL-6 receptor) showed a significant safety signal (ROR = 3.04, 95% CI: 2.67–3.46). T-cell inhibitor abatacept (ROR = 3.33, 95% CI: 2.96–3.76) and JAK inhibitors upadacitinib (ROR = 2.51, 95% CI: 2.03–3.09), baricitinib (ROR = 2.90, 95% CI: 1.61–5.25), and tofacitinib (ROR = 1.32, 95% CI: 1.13–1.54) also demonstrated positive signals. These findings were consistent with the information component analysis (Table 2).

**TABLE 2:** Full database-wide disproportionality analysis of safety signals for 36 disease-modifying antirheumatic drugs in FAERS.

Type of DMARDs	Case reports	ROR (95% CI)	IC (IC_025_)
Conventional DMARDs			
Methotrexate	848	5.43 (5.07–5.82)	2.38 (2.27)
Leflunomide	169	4.31 (3.70–5.01)	2.08 (1.83)
Sulfasalazine	44	5.65 (4.20–7.60)	2.42 (1.92)
Hydroxychloroquine	68	3.30 (2.60–4.19)	1.69 (1.29)
Azathioprine	10	1.49 (0.80–2.77)	0.54 (−0.54)
Cyclosporine	50	0.87 (0.66–1.15)	−0.20 (−0.67)
Mycophenolate mofetil	97	2.05 (1.68–2.51)	1.03 (0.69)
Cyclophosphamide	59	2.13 (1.65–2.76)	1.08 (0.65)
Penicillamine	1	1.92 (0.27–13.65)	0.56 (−3.23)
Sodium aurothiomalate	0	NA	−0.05 (−10.38)
Auranofin	0	NA	−0.10 (−10.42)
TNF-α-targeted inhibitors			
Adalimumab	809	1.26 (1.18–1.35)	0.32 (0.21)
Etanercept	729	1.55 (1.44–1.67)	0.61 (0.49)
Infliximab	279	1.20 (1.07–1.35)	0.26 (0.06)
Golimumab	71	1.97 (1.56–2.49)	0.97 (0.57)
Certolizumab pegol	109	1.63 (1.35–1.96)	0.69 (0.38)
B-cell inhibitors			
Belimumab	10	0.46 (0.25–0.86)	−1.07 (−2.15)
Rituximab	583	4.14 (3.82–4.50)	2.01 (1.87)
Interleukin inhibitors			
Anakinra	7	0.68 (0.33–1.43)	−0.52 (−1.82)
Canakinumab	3	0.33 (0.11–1.03)	−1.44 (−3.51)
Guselkumab	3	0.23 (0.07–0.70)	−1.97 (−4.04)
Ixekizumab	3	0.17 (0.06–0.54)	−2.33 (−4.40)
Risankizumab	28	0.95 (0.66–1.38)	−0.07 (−0.70)
Sarilumab	7	0.70 (0.33–1.47)	−0.48 (−1.79)
Secukinumab	76	0.60 (0.48–0.75)	−0.73 (−1.11)
Tocilizumab	237	3.04 (2.67–3.46)	1.58 (1.37)
Ustekinumab	42	0.81 (0.59–1.09)	−0.31 (−0.82)
Tildrakizumab	0	NA	−1.20 (−11.53)
Brodalumab	0	NA	−2.25 (−12.58)
T-cell inhibitors			
Abatacept	269	3.33 (2.96–3.76)	1.71 (1.51)
JAK inhibitors			
Upadacitinib	87	2.51 (2.03–3.09)	1.31 (0.95)
Baricitinib	11	2.90 (1.61–5.25)	1.42 (0.40)
Tofacitinib	161	1.32 (1.13–1.54)	0.40 (0.14)
Iguratimod	0	NA	−0.01 (−10.33)
Filgotinib	0	NA	−0.02 (−10.34)
PDE4-targeted inhibitor			
Apremilast	19	0.22 (0.14–0.34)	−2.15 (−2.92)

DMARDs, disease-modifying antirheumatic drugs; ROR, reporting odds ratio; 95%CI, the 95% confidence interval; IC, information component; IC025, the lower limit of the 95% credibility interval of the information component; JAK, janus kinase; TNF, tumor necrosis factor; NA, not available due to small sample size (n < 3).

### Active-comparator restricted disproportionality analysis in FAERS

**FIGURE 4:**
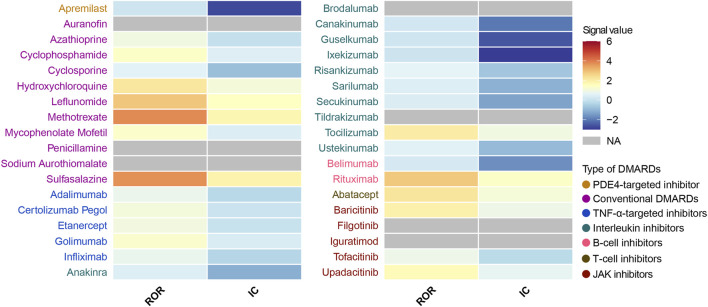
Heatmap presents active-comparator restricted disproportionality results of pulmonary fibrosis safety signals for 36 DMARDs in FAERS. *NA* indicates that the number of reported cases is fewer than 3. DMARDs, disease-modifying antirheumatic drugs; ROR, reporting odds ratio; IC, information component; TNF, tumor necrosis factor; JAK, Janus kinase. NA, not available.

Next, we conducted an active-comparator restricted disproportionality analysis within a specific population of patients receiving DMARDs or reporting comorbid autoimmune diseases (Figure 4). The pulmonary fibrosis signal generally decreased, but certain DMARDs still exhibited significantly over-reported events. CsDMARDs including methotrexate, leflunomide, sulfasalazine, hydroxychloroquine, mycophenolate mofetil, and cyclophosphamide showed ROR values ranging from 1.35 to 3.80. Among TNF-α inhibitors, golimumab showed a significantly elevated ROR for pulmonary fibrosis (ROR = 1.29, 95% CI: 1.02–1.63); however, the information component analysis did not meet the predefined signal threshold (IC = 0.36, IC025 = −0.03), and therefore this finding should not be interpreted as a confirmed signal. Similar findings were observed with baricitinib in JAK inhibitors. Biologic DMARDs rituximab, tocilizumab, abatacept, and upadacitinib remained to show significantly over-reported pulmonary fibrosis events, with ROR values ranging from 1.64 to 2.81 (Table 3).

**TABLE 3:** Active-comparator restricted disproportionality analysis of safety signals for 36 disease-modifying antirheumatic drugs in FAERS.

Type of DMARDs	Case reports	ROR (95% CI)	IC (IC_025_)
Conventional DMARDs			
Methotrexate	848	3.80 (3.53–4.08)	1.77 (1.66)
Leflunomide	169	2.85 (2.44–3.32)	1.48 (1.22)
Sulfasalazine	44	3.71 (2.76–4.99)	1.84 (1.34)
Hydroxychloroquine	68	2.17 (1.71–2.75)	1.09 (0.69)
Azathioprine	10	0.97 (0.52–1.81)	−0.04 (−1.11)
Cyclosporine	50	0.57 (0.43–0.75)	−0.81 (−1.28)
Mycophenolate mofetil	97	1.35 (1.10–1.64)	0.42 (0.08)
Cyclophosphamide	59	1.40 (1.08–1.81)	0.47 (0.04)
Penicillamine	1	1.92 (0.27–13.65)	0.56 (−3.23)
Sodium aurothiomalate	0	NA	−0.05 (−10.38)
Auranofin	0	NA	−0.10 (−10.42)
TNF-α-targeted inhibitors			
Adalimumab	809	0.79 (0.73–0.85)	−0.29 (−0.41)
Etanercept	729	1.00 (0.93–1.08)	0 (−0.12)
Infliximab	279	0.77 (0.69–0.87)	−0.35 (−0.55)
Golimumab	71	1.29 (1.02–1.63)	0.36 (−0.03)
Certolizumab pegol	109	1.06 (0.88–1.28)	0.09 (−0.23)
B-cell inhibitors			
Belimumab	10	0.30 (0.16–0.56)	−1.68 (−2.75)
Rituximab	583	2.81 (2.58–3.06)	1.40 (1.26)
Interleukin inhibitors			
Anakinra	7	0.45 (0.21–0.94)	−1.11 (−2.41)
Canakinumab	3	0.22 (0.07–0.67)	−2.03 (−4.10)
Guselkumab	3	0.15 (0.05–0.46)	−2.57 (−4.63)
Ixekizumab	3	0.11 (0.04–0.35)	−2.93 (−5.00)
Risankizumab	28	0.62 (0.43–0.90)	−0.68 (−1.31)
Sarilumab	7	0.46 (0.22–0.96)	−1.07 (−2.37)
Secukinumab	76	0.38 (0.31–0.48)	−1.34 (−1.72)
Tocilizumab	237	2.01 (1.76–2.29)	0.98 (0.76)
Ustekinumab	42	0.52 (0.39–0.71)	−0.92 (−1.43)
Tildrakizumab	0	NA	−1.20 (−11.53)
Brodalumab	0	NA	−2.25 (−12.58)
T-cell inhibitors			
Abatacept	269	2.21 (1.96–2.50)	1.11 (0.90)
JAK inhibitors			
Upadacitinib	87	1.64 (1.33–2.03)	0.70 (0.35)
Baricitinib	11	1.90 (1.05–3.44)	0.87 (−0.15)
Tofacitinib	161	0.86 (0.73–1.00)	−0.21 (−0.47)
Iguratimod	0	NA	−0.01 (−10.33)
Filgotinib	0	NA	−0.02 (−10.34)
PDE4-targeted inhibitor			
Apremilast	19	0.14 (0.09–0.22)	−2.76 (−3.53)

DMARDs, disease-modifying antirheumatic drugs; ROR, reporting odds ratio; 95%CI, the 95% confidence interval; IC, information component; IC025, the lower limit of the 95% credibility interval of the information component; JAK, janus kinase; TNF, tumor necrosis factor; NA, not available due to small sample size (n < 3).

### Disproportionality analysis in VigiAccess

**FIGURE 5:**
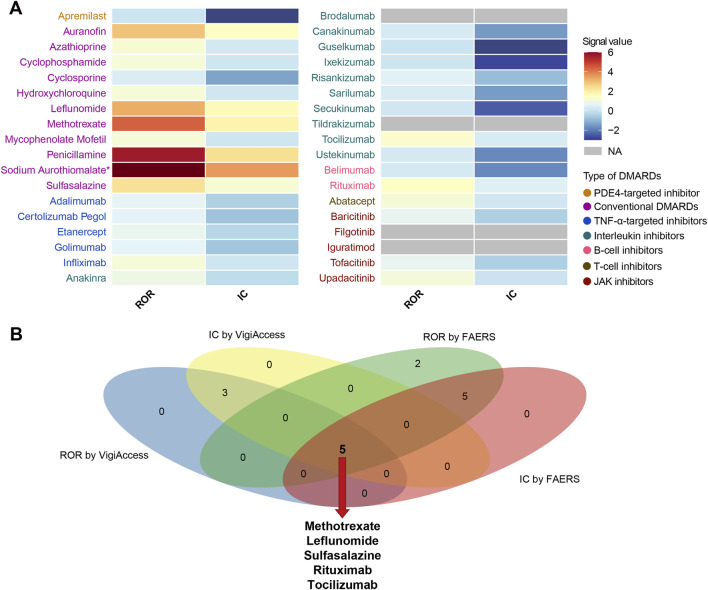
Pulmonary fibrosis safety signals for 36 DMARDs in VigiAccess and comparison with results from FAERS. **(A)** Heatmap presents active-comparator restricted disproportionality analysis of safety signals for 36 DMARDs in VigiAccess. **(B)** Venn diagram shows the overlap of drug-specific pulmonary fibrosis signals identified by different pharmacovigilance algorithms in both databases. DMARDs, disease-modifying antirheumatic drugs; FAERS, Food and Drug Administration’s Adverse Event Reporting System; ROR, reporting odds ratio; IC, information component; TNF, tumor necrosis factor; JAK, Janus kinase; NA, not available. *Indicates that the ROR for Sodium Aurothiomalate is 13.87, which exceeds the color scale range.

We analyzed the DMARDs-related pulmonary fibrosis using summary-level data from VigiAccess (Figure 5A). Among 36 DMARDs, eight DMARDs showed significantly elevated reporting signals for pulmonary fibrosis. Specifically, the csDMARDs included methotrexate (ROR = 4.39, 95% CI: 4.11–4.70), leflunomide (ROR = 3.26, 95% CI: 2.75–3.86), sulfasalazine (ROR = 2.33, 95% CI: 1.91–2.84), penicillamine (ROR = 5.67, 95% CI: 3.98–8.08), sodium aurothiomalate (ROR = 13.87, 95% CI: 10.20–18.84), and auranofin (ROR = 2.90, 95% CI: 1.56–5.40) (Table 4). Additionally, B-cell inhibitor rituximab (ROR = 1.43, 95% CI: 1.27–1.61) and IL-6R inhibitor tocilizumab (ROR = 1.28, 95% CI: 1.08–1.51) also exhibited notable over-reporting signals.

**TABLE 4:** Active-comparator restricted disproportionality analysis of safety signals for 36 disease-modifying antirheumatic drugs in VigiAccess.

Type of DMARDs	Case reports	ROR (95% CI)	IC (IC_025_)
Conventional DMARDs			
Methotrexate	1,097	4.39 (4.11–4.70)	1.85 (1.75)
Leflunomide	138	3.26 (2.75–3.86)	1.66 (1.38)
Sulfasalazine	101	2.33 (1.91–2.84)	1.19 (0.86)
Hydroxychloroquine	66	1.14 (0.90–1.46)	0.19 (−0.22)
Azathioprine	63	1.20 (0.93–1.54)	0.25 (−0.16)
Cyclosporine	40	0.38 (0.28–0.52)	−1.35 (−1.87)
Mycophenolate mofetil	121	1.11 (0.93–1.33)	0.15 (−0.15)
Cyclophosphamide	244	1.11 (0.98–1.27)	0.15 (−0.07)
Penicillamine	31	5.67 (3.98–8.08)	2.38 (1.79)
Sodium aurothiomalate	42	13.87 (10.20–18.84)	3.56 (3.04)
Auranofin	10	2.90 (1.56–5.40)	1.41 (0.33)
TNF-α-targeted inhibitors			
Adalimumab	782	0.67 (0.62–0.73)	−0.46 (−0.58)
Etanercept	663	0.77 (0.71–0.84)	−0.31 (−0.44)
Infliximab	319	1.12 (1.00–1.25)	0.15 (−0.03)
Golimumab	50	0.62 (0.47–0.82)	−0.67 (−1.14)
Certolizumab pegol	72	0.59 (0.47–0.75)	−0.73 (−1.13)
B-cell inhibitors			
Belimumab	9	0.28 (0.15–0.54)	−1.76 (−2.90)
Rituximab	287	1.43 (1.27–1.61)	0.49 (0.29)
Interleukin inhibitors			
Anakinra	13	0.89 (0.52–1.53)	−0.16 (−1.10)
Canakinumab	4	0.33 (0.12–0.88)	−1.48 (−3.25)
Guselkumab	4	0.11 (0.04–0.29)	−3.06 (−4.82)
Ixekizumab	8	0.13 (0.07–0.27)	−2.80 (−4.01)
Risankizumab	40	0.54 (0.40–0.74)	−0.87 (−1.40)
Sarilumab	8	0.35 (0.18–0.70)	−1.45 (−2.66)
Secukinumab	37	0.17 (0.13–0.24)	−2.45 (−3.00)
Tocilizumab	142	1.28 (1.08–1.51)	0.35 (0.07)
Ustekinumab	34	0.29 (0.21–0.40)	−1.75 (−2.32)
Tildrakizumab	0	NA	−2.77 (−13.10)
Brodalumab	1	0.23 (0.03–1.66)	−1.67 (−5.45)
T-cell inhibitors			
Abatacept	145	1.15 (0.98–1.36)	0.20 (−0.08)
JAK inhibitors			
Upadacitinib	92	1.07 (0.87–1.31)	0.09 (−0.25)
Baricitinib	21	0.71 (0.46–1.10)	−0.47 (−1.21)
Tofacitinib	142	0.71 (0.60–0.84)	−0.47 (−0.75)
Iguratimod	0	NA	−2.07 (−12.39)
Filgotinib	2	0.79 (0.20–3.15)	−0.28 (−2.88)
PDE4-targeted inhibitor			
Apremilast	19	0.10 (0.07–0.16)	−3.19 (−3.96)

DMARDs, disease-modifying antirheumatic drugs; ROR, reporting odds ratio; 95%CI, the 95% confidence interval; IC, information component; IC025, the lower limit of the 95% credibility interval of the information component; JAK, janus kinase; TNF, tumor necrosis factor; NA, not available due to small sample size (n < 3).

Combining the results from both FAERS and VigiAccess databases, five DMARDs, including methotrexate, leflunomide, sulfasalazine, rituximab, and tocilizumab, consistently showed significantly increased reporting of pulmonary fibrosis events (Figure 5B).

### Risk factor analysis

**FIGURE 6:**
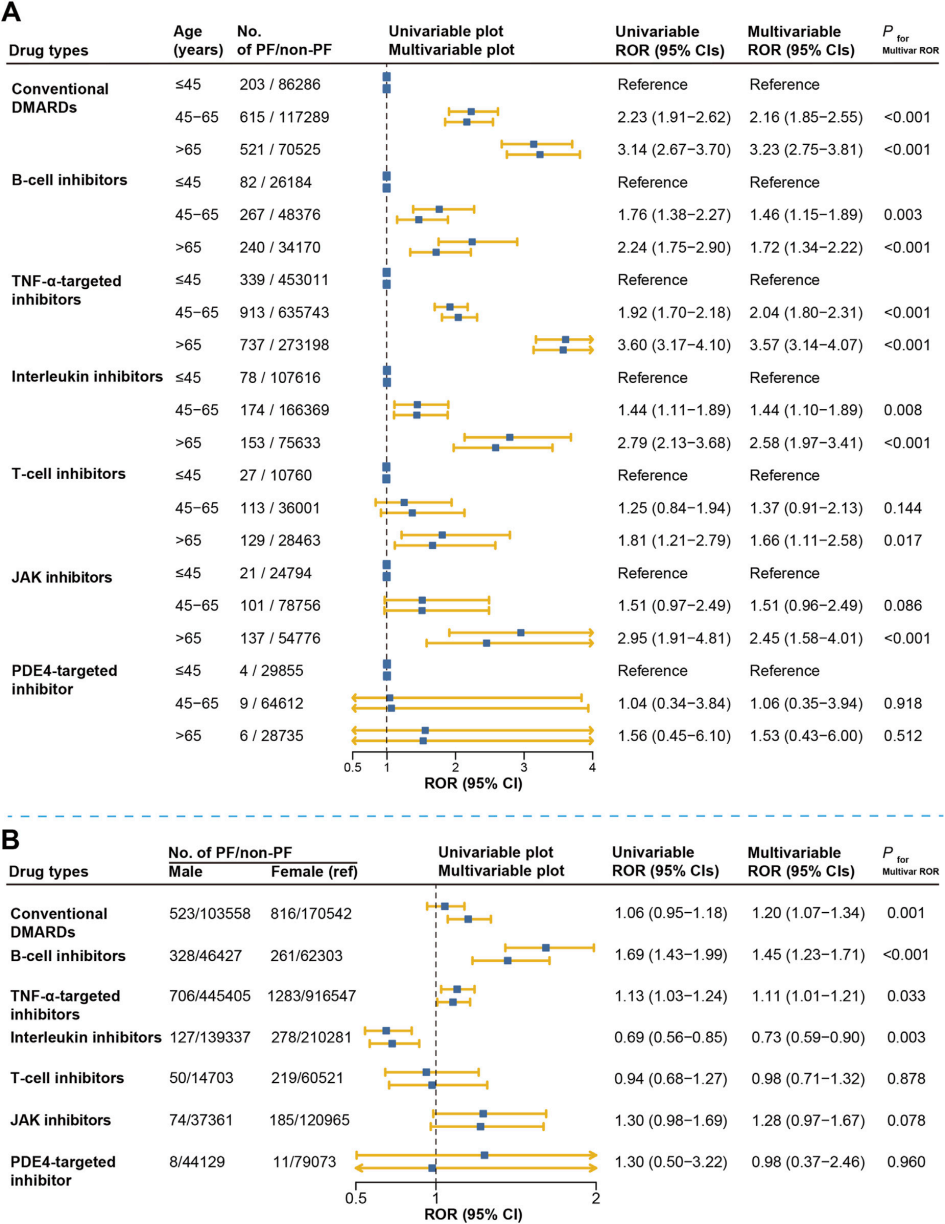
Risk factor analysis for DMARDs-related pulmonary fibrosis reporting in FAERS. **(A)** represents the results of disproportionate comparison of pulmonary fibrosis according to different age groups. **(B)** represents the results of disproportionate comparison of pulmonary fibrosis according to gender groups. Multivariable logistic regression was adjusted for reporting year, region, age (in the sex analysis), and sex (in the age analysis). DMARDs, disease-modifying antirheumatic drugs; ROR, reporting odds ratio; TNF, tumor necrosis factor; JAK, Janus kinase; NA, not available.

We further examined age and sex as potential risk factors associated with DMARD-related pulmonary fibrosis. We found that older age appears to be a strong risk factor for each DMARDs type, except in the PDE4-targeted inhibitor where there was no statistical significance. Compared to patients younger than 45, those aged 45–65 and those older than 65 showed significantly higher reporting rates for pulmonary fibrosis, both in univariate and multivariate logistic regression analyses. For instance, among conventional DMARD users, patients aged 45–65 and those older than 65 had approximately double and triple the reporting rates, respectively, compared with users <45 years of age (ROR _adjusted_ = 2.16, 95% CI: 1.85–2.55; ROR _adjusted_ = 3.23, 95% CI: 2.75–3.81) (Figure 6A).

Regarding sex differences, we observed higher reporting rates of pulmonary fibrosis among male users of conventional DMARDs, B-cell inhibitors, and TNF-α inhibitors, with adjusted ROR ranging from 1.20 to 1.45 compared to females. Similarly, male patients treated with JAK inhibitors tended to have a higher reporting rate (ROR _adjusted_ = 1.28, 95% CI: 0.97–1.67), though statistically non-significant (*P* = 0.078). In contrast, among users of interleukin inhibitors, males had significantly lower reporting rates compared to females (ROR _adjusted_ = 0.73, 95% CI: 0.59–0.90). No significant sex difference was found in the T-cell or PDE4-targeted inhibitor group (Figure 6B).

### Time-to-onset analysis

**FIGURE 7:**
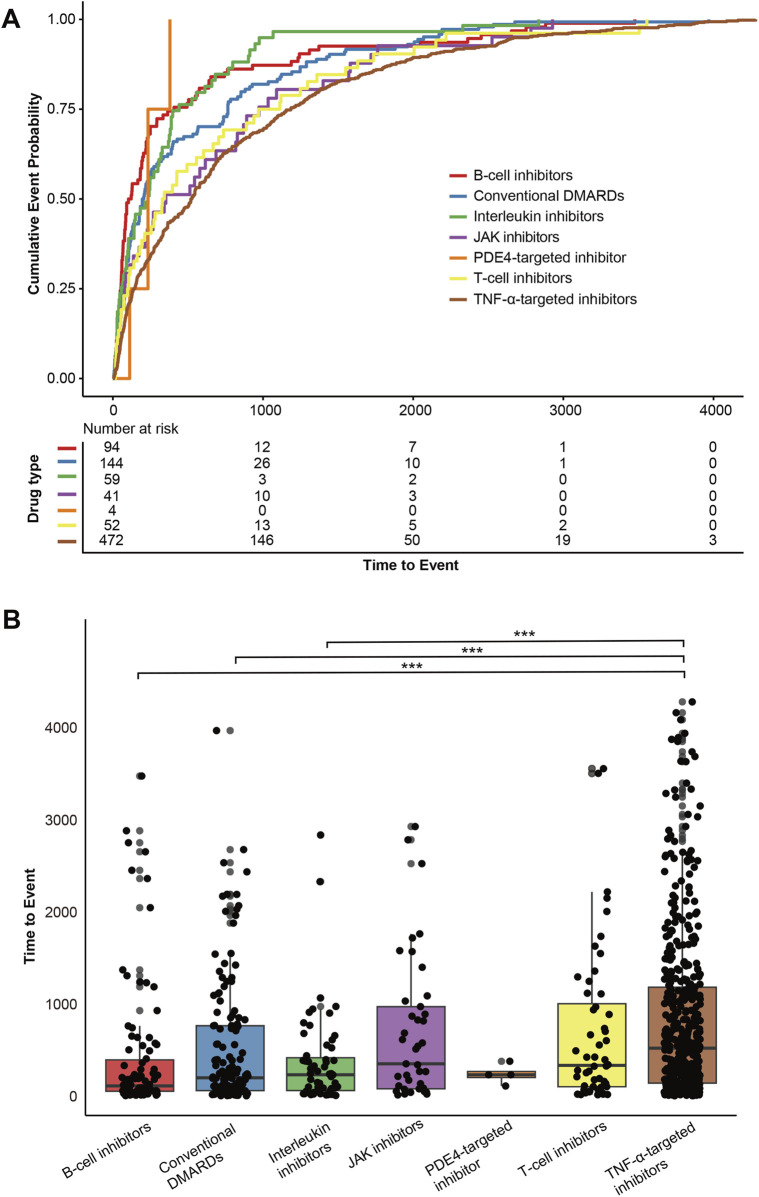
Time-to-onset analysis of DMARDs-related pulmonary fibrosis in FAERS. **(A)** Cumulative event probability curves for the time-to-onset of pulmonary fibrosis across different DMARD categories. **(B)** Dunn’s post-hoc multiple comparison test for time-to-onset across different DMARD categories, with Bonferroni correction applied. *<0.05, **<0.01, ***<0.001.

Our analysis revealed notable differences in the timing of pulmonary fibrosis onset across DMARD categories (Figure 7A). B-cell inhibitors showed the shortest median time-to-onset (113 days, IQR: 54–397), followed by conventional DMARDs (201 days, IQR: 61–766), interleukin inhibitors (234 days, IQR: 62–419), PDE4-targeted inhibitor (234 days, IQR: 204–271), T-cell inhibitors (336 days, IQR: 103–1,006), JAK inhibitors (353 days, IQR: 81–973), and TNF-α inhibitors (523 days, IQR: 143–1,185). The Kruskal-Wallis rank-sum test confirmed significant differences across DMARD categories (*P* < 0.001). Dunn’s multiple comparison tests with Bonferroni correction were performed, and detailed results are presented in Figure 7B.

## Discussion

To the best of our knowledge, this is the first large-scale pharmacovigilance analysis to systematically investigate the association between different DMARDs and pulmonary fibrosis. We identified five DMARDs, including methotrexate, leflunomide, sulfasalazine, rituximab, and tocilizumab, that consistently showed significant over-reporting of pulmonary fibrosis events in both FAERS and VigiAccess. These findings provide a pharmacovigilance perspective on the long-standing concerns regarding DMARDs-related pulmonary fibrosis. Our results indicate that only a subset of DMARDs, rather than all, are associated with over-reported pulmonary fibrosis events. Notably, age emerged as a strong risk factor for pulmonary fibrosis in DMARD users, while the influence of sex varied depending on the specific DMARD. These findings are valuable for developing more personalized strategies for monitoring pulmonary fibrosis.

Concerns about the potential link between DMARDs and pulmonary fibrosis have existed for decades, yet prior evidence has largely come from small-scale case reports. Several csDMARDs, such as leflunomide, methotrexate, and azathioprine, have been associated with interstitial lung disease in some studies. Several research suggests that csDMARDs may induce or exacerbate interstitial lung disease in rheumatoid arthritis patients and accelerate lung function decline (Khadadah et al., 2002; Kamata et al., 2004; Ochi et al., 2006; Savage et al., 2006). A meta-analysis by Conway et al. found a slight but significant increase in the association between methotrexate and overall respiratory adverse events (Conway et al., 2014). However, there are conflicting findings, such as studies by Kiely et al. and Juge et al. found that methotrexate neither increased the incidence of interstitial lung disease nor accelerated its onset in rheumatoid arthritis patients (Kiely et al., 2019; Juge et al., 2021). Some other studies have also failed to observe a positive association between methotrexate and interstitial lung disease (Fragoulis et al., 2019). However, most of these studies did not focus specifically on pulmonary fibrosis, which is a rare but severe adverse event that represents a smaller proportion of interstitial lung disease cases. As a result, previous evidence has struggled to quantify or confirm the impact of these DMARDs on pulmonary fibrosis. Moreover, studies based on case reports may be subject to bias due to random observations and confounding factors. Recent interventional studies in pulmonary fibrosis further underscore the high morbidity, mortality, and limited therapeutic options associated with this condition (Li et al., 2023), highlighting the importance of early detection and prevention. In present study, large-scale pharmacovigilance analyses support pulmonary fibrosis as a significant safety signal for methotrexate, leflunomide, and sulfasalazine, although causality still cannot be established.

A similar knowledge gap exists for bDMARDs. Compared to conventional DMARDs, more case reports associated bDMARDs directly with pulmonary fibrosis. In 2006, Huggett et al. and Schoe et al. reported cases of pulmonary fibrosis in elderly rheumatoid arthritis patients after the use of adalimumab, with both patients having normal chest X-rays prior to treatment (Huggett and Armstrong, 2006; Schoe et al., 2006). Cohen et al. described a case of a young psoriatic arthritis patient who developed diffuse interstitial pulmonary fibrosis shortly after adalimumab treatment and died within a month (Cohen et al., 2011). Additional reports also indicate that bDMARDs, such as adalimumab, infliximab, and etanercept, may cause rapid worsening of pulmonary fibrosis, even leading to fatal outcomes in some patients (Ostor et al., 2004; Allanore et al., 2006; Tournadre et al., 2008). However, it remains unclear whether the observed pulmonary fibrosis was incidental or worsened by treatment. In addition, little is known about tsDMARDs-related pulmonary fibrosis. In the current study, we quantified the disproportionate reporting rates of pulmonary fibrosis for each DMARD using pharmacovigilance algorithms. Interestingly, in the active comparators-restricted analysis, adalimumab, infliximab, and etanercept did not show disproportionate increases in pulmonary fibrosis events. While rituximab and tocilizumab consistently demonstrated positive signals in both FAERS and VigiAccess, suggesting that these drugs require more careful evaluation of pulmonary fibrosis in clinical practice.

Our study found that age is a significant risk factor for pulmonary fibrosis across various types of DMARDs. This finding supports the recommendation for baseline lung function assessment before initiating DMARD treatment in elderly populations, along with enhanced monitoring during treatment. Regarding sex differences, we observed that males had a higher incidence of pulmonary fibrosis in certain DMARD categories, including csDMARDs, TNF-α inhibitors, and B-cell inhibitors. However, female patients were at greater risk when treated with interleukin inhibitors. This suggests that gender-specific monitoring and risk assessment strategies may be necessary when prescribing different targeted therapies. These results also suggest that clinicians may consider closer monitoring for pulmonary symptoms and imaging abnormalities in high-risk populations and be more cautious in selecting drugs with stronger safety signals. Such measures may facilitate earlier recognition of drug-related pulmonary fibrosis, timely intervention, and potentially improved patient outcomes. The mechanisms behind this phenomenon likely involve gender-related differences in immune regulation and metabolic characteristics, which warrant further investigation.

Although our study reveals associations between certain DMARDs and pulmonary fibrosis, the underlying mechanisms remain difficult to elucidate. Recent studies suggest that immune cells and cytokines, including T cells, B cells, TNF-α, and IL-6, play significant roles in the development and progression of pulmonary fibrosis, involving processes such as fibrosis remodeling, alveolar consolidation, and inflammatory activation (Ali et al., 2021; Maranatha et al., 2022; Prêle et al., 2022; Han et al., 2023; Mendoza et al., 2023; Adel et al., 2024; Saha and Talwar, 2024). However, the actual impact of DMARDs in regulating these targets is complex. Interestingly, we observed that some DMARDs-related pulmonary fibrosis might arise from off-target effects. For example, despite both targeting IL-6 receptor, sarilumab and tocilizumab displayed significantly different disproportionate reporting signals for pulmonary fibrosis. Another example involves belimumab and rituximab, which target B cells via B-lymphocyte stimulator and CD20, respectively. Although both drugs reduce B cell levels, their associations with pulmonary fibrosis differ significantly. These inconsistencies indicate that differences in molecular structure, pharmacokinetic properties, or patient usage context may result in varying risks. These findings highlight the complexity and multifactorial nature of pulmonary fibrosis as a potential severe adverse event for DMARDs.

Our study provides valuable data on DMARDs-related pulmonary fibrosis based on two major global pharmacovigilance databases, but there are some important limitations. First, while FAERS and VigiAccess contain a large number of global case reports, these data are primarily derived from spontaneous reporting systems and may be subject to reporting bias. The sources of information are diverse, and the probability of suspecting an adverse reaction to be drug-related is not the same in all cases. Less severe pulmonary fibrosis cases may be underreported, while severe cases may be over-reported. Furthermore, case reports often lack detailed clinical data, such as baseline disease status, comorbidities, lung function, and other potential confounding factors, which could influence the observed associations between drugs and pulmonary fibrosis. Importantly, pulmonary fibrosis can also occur as a manifestation of the underlying autoimmune disease itself, such as rheumatoid arthritis, which poses a key challenge in distinguishing disease-related from drug-induced pulmonary toxicity within pharmacovigilance data. In addition, co-prescriptions such as corticosteroids, DMARDs combination, or antifibrotic therapies are generally not captured in these reports, and their potential interactive impact on pulmonary outcomes cannot be excluded. In our analysis, we excluded cases involving concomitant antifibrotic agents used to treat pulmonary fibrosis, including nintedanib and pirfenidone, to reduce confounding from pre-existing disease. Second, although we used an active-comparator restricted analysis to minimize confounding factors, this remains a signal detection method, not a tool for causal inference. Third, other potential confounders, such as patient characteristics, treatment regimens, and drug history, could affect the pulmonary toxicity risk of DMARDs, but due to data limitations, we could not fully account for these factors. Finally, detailed information on DMARD dosages and treatment duration was not available. This lack of dosing data prevents evaluation of potential dose-response relationships or cumulative exposure effects, both of which may play an important role in the development of drug-induced pulmonary toxicity. Therefore, our findings should be interpreted as safety signals for DMARDs rather than definitive evidence of causality.

## Conclusion

This study systematically explored the safety signals and characteristics of pulmonary fibrosis related to various DMARDs using large-scale pharmacovigilance databases. We provided evidence linking specific DMARDs to an increased reporting of pulmonary fibrosis and identified older age as a strong risk factor. These findings could contribute valuable insights for clinical decision-making regarding drug selection, patient risk assessment, and early monitoring of pulmonary fibrosis. Additionally, our study lays a foundation for future research, including experimental studies in animal models to elucidate the underlying mechanisms and prospective population-based cohort studies to validate the causality of the observed safety signals.

## Data Availability

The original contributions presented in the study are included in the article/supplementary material, further inquiries can be directed to the corresponding author.
